# Effects of changes in residential fast-food outlet exposure on Body Mass Index change: longitudinal evidence from 92,211 Lifelines participants

**DOI:** 10.1186/s12966-024-01577-8

**Published:** 2024-03-14

**Authors:** Carel-Peter L. van Erpecum, Sander K.R. van Zon, Ute Bültmann, Nynke Smidt

**Affiliations:** 1 Department of Epidemiology, University of Groningen, University Medical Center Groningen, Hanzeplein 1, 9700 RB Groningen, The Netherlands; 2 Department of Health Sciences, Community and Occupational Medicine, University of Groningen, University Medical Center Groningen, Hanzeplein 1, 9700 RB Groningen, The Netherlands

**Keywords:** Fast-food outlet, Body Mass Index, Age, Natural experiment

## Abstract

**Background:**

Evidence on the association between fast-food outlet exposure and Body Mass Index (BMI) remains inconsistent and is primarily based on cross-sectional studies. We investigated the associations between changes in fast-food outlet exposure and BMI changes, and to what extent these associations are moderated by age and fast-food outlet exposure at baseline.

**Methods:**

We used 4-year longitudinal data of the Lifelines adult cohort (*N* = 92,211). Participant residential addresses at baseline and follow-up were linked to a register containing fast-food outlet locations using geocoding. Change in fast-food outlet exposure was defined as the number of fast-food outlets within 1 km of the residential address at follow-up minus the number of fast-food outlets within 1 km of the residential address at baseline. BMI was calculated based on objectively measured weight and height. Fixed effects analyses were performed adjusting for changes in covariates and potential confounders. Exposure-moderator interactions were tested and stratified analyses were performed if *p* < 0.10.

**Results:**

Participants who had an *increase* in the number of fast-food outlets within 1 km had a greater BMI increase (B(95% CI): 0.003 (0.001,0.006)). Decreases in fast-food outlet exposure were not associated with BMI change (B(95% CI): 0.001 (-0.001,0.004)). No clear moderation pattern by age or fast-food outlet exposure at baseline was found.

**Conclusions:**

Increases in residential fast-food outlet exposure are associated with BMI gain, whereas *decreases* in fast-food outlet exposure are not associated with BMI loss. Effect sizes of increases in fast-food outlet exposure on BMI change were small at individual level. However, a longer follow-up period may have been needed to fully capture the impact of increases in fast-food outlet exposure on BMI change. Furthermore, these effect sizes could still be important at population level considering the rapid rise of fast-food outlets across society. Future studies should investigate the mechanisms and changes in consumer behaviours underlying associations between changes in fast-food outlet exposure and BMI change.

**Supplementary Information:**

The online version contains supplementary material available at 10.1186/s12966-024-01577-8.

## Introduction

The obesogenic environment, especially exposure to fast-food outlets, is increasingly recognised as an explanation for the rise in overweight and obesity in the global adult population [1]. Fast-food outlets can be characterised as outlets that are easily accessible [1], have long opening hours, and serve highly caloric, unhealthy meals [2]. The number of fast-food outlets has increased substantially over the past years [3]. In the Netherlands, the number of fast-food outlets increased from 14,625 outlets in 2016 to 18,521 outlets in 2021, an increase of 27% [4].

To date, evidence on the association between fast-food outlet exposure and Body Mass Index (BMI) in adults remains inconsistent [5–10]. The evidence is predominantly based on cross-sectional data, which precludes the estimation of the *causal* effects of fast-food outlet exposure in BMI. Randomised controlled trials, the gold standard for investigating causal effects in epidemiology, are not feasible, as it is not possible to randomly assign individuals to different living environments. As an alternative approach towards causality, researchers have investigated changes in fast-food outlet exposure, e.g. openings or closings of fast-food outlets and moving houses to an area with a different level of fast-food outlet exposure [11], in relation to BMI change. These changes in fast-food outlet exposure are then assumed to exert an effect on BMI because they change the barrier for fast-food consumption, which has consistently been linked to weight gain [12]. The effect of changes in fast-food outlet exposure on BMI is likely to be visible only after several years, as health behavioural models (e.g. the Transtheoretical model of Behavior Change [13]) suggest that health behaviours such as fast-food consumption only change slowly over time. Examining changes in fast-food outlet exposure may elucidate the causal role of fast-food outlet exposure in BMI change and inform policies that target the fast-food environment (e.g., restricting openings of new fast-food outlets).

Studies provide mixed support for the hypothesis that changes in fast-food outlet exposure are associated with BMI changes in adults. Zenk and colleagues [14] found that changes in the number of fast-food outlets within 1 and 3 miles (1.6 kilometre (km) and 3.2 km) from the residential address were weakly but significantly associated with a 0.025 kg/m^2^ BMI increases over 5 years among 1.7 million United States veterans. However, these findings were not confirmed in other studies [15–21]. Importantly, the evidence base is primarily focused on the United States, where the structure of the built environment and mobility patterns differ from Europe. Furthermore, studies contain several methodological challenges. For instance, some studies measured fast-food outlet exposure at neighbourhood level instead of individual level [18] or used self-reported BMI instead of objectively measured BMI [18, 20], introducing the risk of information bias. Moreover, part of the studies could not extensively adjust for socio-demographic characteristics [14, 17] or environmental factors [15–17, 19, 21], and all studies contained samples not fully representative of the general population.

Moreover, associations between changes in fast-food outlet exposure and BMI change may be moderated by age and the baseline level of fast-food outlet exposure. Young adults consume fast-food relatively often [22, 23] and increase more in BMI than adults in later life stages [24]. However, to our best knowledge, no previous study investigated the potentially moderating role of age in the association between changes in fast-food outlet exposure and BMI change. Furthermore, we reason that associations between changes in fast-food outlet exposure and BMI change could be stronger when the baseline level of fast-food outlet exposure is low (e.g., having null fast-food outlets in the area) than high (e.g., having ten fast-food outlets in the area). A study on Dutch children found that increases in fast-food outlet exposure were only associated with greater BMI increases when there were no fast-food outlets around within 400 metres (m) of the residential address at baseline [25]. To our best knowledge, studies in adults did not take into account the potentially moderating role of the baseline level of fast-food outlet exposure in the associations between changes in fast-food outlet exposure and BMI change.

We investigated the associations between changes in residential fast-food outlet exposure and BMI change over a 4-year period among the Dutch general adult population. Additionally, we investigated moderation by age and fast-food outlet exposure at baseline within these associations. We hypothesized that changes in fast-food outlet exposure are positively associated with BMI change, and that these associations are stronger in young adulthood and with a low baseline level of fast-food outlet exposure.

## Methods

### Study population

We used baseline (November 2006-December 2013) and four-year follow-up data (January 2014-December 2017) from adults of the Lifelines Cohort Study [26]. Lifelines is a prospective population-based cohort study examining in a unique three-generational design the health and health-related behaviours of 167,729 persons living in the North of the Netherlands. It employs a broad range of investigative procedures in assessing the biomedical, socio-demographic, behavioural, physical and psychological factors, which contribute to the health and disease of the general population, with a special focus on multi-morbidity and complex genetics. Participants were recruited through general practitioners, family members of participants, and online registrations. Lifelines participants are broadly representative of the Northern Netherlands adult general population in terms of socio-economic characteristics, lifestyle factors, prevalence of chronic diseases, and general health [27]. Using nationwide address registry data, participant baseline and follow-up assessment residential addresses were obtained and geo-coded.

In the current study, we excluded [1] participants residing in a nursing home at baseline or at some point between baseline and follow-up (*N* = 324), because they may not always have been able to interact with their fast-food environment, [2] women who were pregnant at or up to a year prior to the baseline assessment or follow-up assessment (*N* = 4,801), as the BMI measurement at the assessments then does not reflect their actual weight status due to the elevated BMI because of the pregnancy, [3] participants who were lost to follow-up (*N* = 40,881), and [4] participants with missing data on fast-food outlet exposure, BMI (either at baseline or follow-up), or in case more than 30% of the covariates and potential confounders were missing (*N* = 14,337) [28]. This cut-off of 30% missing data on covariates and potential confounders was based on previous research [28] which suggested that having too many missing data on covariates and potential confounders may not provide sufficient participant information for multiple imputation.

### Data linkage

We linked participants’ geo-coded residential addresses at baseline and follow-up to LISA data (www.lisa.nl), a Dutch register containing locations where paid work is performed for at least one hour/month. The validity of the LISA data has been confirmed elsewhere [29]. Residential addresses at baseline and follow-up were linked to LISA data of 2012 and 2015, respectively, matching the median recruitment years of these assessment rounds. Importantly, the large majority of participants had their baseline and follow-up assessment less than a year from 2012 and 2015, respectively (70.2% had the baseline assessment between 2011 and 2013 and 86.3% had the follow-up assessment between 2014 and 2016). We then extracted locations of fast-food outlets, physical activity facilities and healthy food outlets from the LISA data using Standard Business Information codes (Table S1 and [30] for definitions). We also linked Lifelines participants’ neighbourhood codes of 2012 and 2015 to Statistics Netherlands neighbourhood data from those years. Neighbourhood boundaries were based on official administrative definitions from Statistics Netherlands [31]. The three northern provinces of the Netherlands (i.e., Groningen, Friesland, and Drenthe) where the Lifelines Cohort Study was conducted contain 1,984 neighbourhoods, which cover a median surface of 156 hectares and contain a median of 616.5 residents.

### Exposure

Based on the linkage with LISA data, we computed the change in number of fast-food outlets within a straight-line 1 km distance around the residential address between baseline and follow-up (i.e. a continuous variable). The 1-km distance was based on a study investigating the association between fast-food outlet exposure and health related outcomes in the Netherlands [32]. We used a separate continuous variable for *increases* in number of fast-food outlets within 1 km and a separate continuous variable for *decreases* in number of fast-food outlets within 1 km, because the associations with BMI change may be stronger for increases than decreases in fast-food outlet exposure. *Increases* in fast-food outlet exposure may result in more cues towards eating fast-food, and subsequently habitual fast-food consumption [33] and BMI increases. However, based on habit formation theory [34], it could be argued that the habit of fast-food consumption may still remain for a substantial period of time when fast-food outlet exposure decreases, and hence these cues towards eating fast-food are taken away. We tested for linearity and observed that the increases in number of fast-food outlets within 1 km and decreases in number of fast-food outlets within 1 km were linearly related to BMI changes.

### Outcome

BMI change between baseline and 4-year follow-up was defined as the difference between BMI at follow-up and BMI at baseline, so that positive numbers indicate increases in BMI. The BMI data at baseline and follow-up were based on objective weight (without shoes and heavy clothing) and height measurements taken by trained research staff at one of the research sites. We used the BMI as this is the most common measure of overweight and obesity due to its easy and inexpensive assessment [35]. Further, as BMI is the outcome in most studies on the fast-food environment, using BMI ensures a more adequate comparison between results of this study and the previous literature.

### Moderators

Age at baseline was categorised as 18–29, 30–39, 40–49, 50–59, and 60 + years. The number of fast-food outlets within 1 km around the residential address at baseline was categorised into null, one, and at least two, based on a previous cross-sectional study on the association between fast-food outlet exposure and BMI in the Netherlands [36].

### Covariates and potential confounders

Individual-level covariates and potential confounders included: follow-up time (in months), weekly working hours; household size (living together or living alone); number of healthy food outlets within 1 km; number of days of at least 30 minutes physical activity (i.e., bicycling, gardening, doing exercise, and doing odds jobs) per week; number of physical activity facilities within 1 km; pregnancy (between the baseline assessment and a year prior to the follow-up assessment); income (net monthly; treated continuously by taking the middle value of categories <€750 (set to €500), €750-€1,000, subsequent €500-intervals until €3,500, and >€3,500 (set to €3,750), divided by the square root of individuals living from that income [37]); and years of education received (based on the highest level of education completed, with less than primary education set to 5 years, primary education set to 6 years, lower or secondary vocational education set to 9 years, junior general secondary education set to 10 years, secondary vocational education, work-based learning pathway, or senior general secondary education set to 12 years, higher vocational education set to 15 years, and university education set to 17 years [38]). Physical activity facilities were included as a covariate or potential confounder as these facilities may co-locate with fast-food outlets and the exposure to physical activity facilities may affect Body Mass Index as as exposure to physical activity facilities may lower the barrier for physical activity behaviours [39]. Neighbourhood-level covariates and potential confounders were address density (number of addresses per km^2^) and neighbourhood socio-economic status based on data of Statistics Netherlands linked to Lifelines participants’ neighbourhood codes. Neighbourhood socio-economic status was measured as a z-standardised composite score using principal component analysis, based on the [1] average value of a house, [2] percentage houses being owner-occupied, [3] mean net disposable monthly income, and [4] percentage of individuals aged 15–65 years receiving assistance benefits, reflecting the financial, occupational and housing situation in a neighbourhood [40].

### Statistical analysis

First, we assessed patterns of missing data through Little’s test, which suggested that the hypothesis of data being Missing Completely at Random (MCAR) was violated (*p* < 0.001). Hence, we imputed missing data through Multiple Imputation by Chained Equations using Multilevel Data (MICEMD) to take into account clustered data at neighbourhood level. We created 10 imputed datasets [41] and all analyses were run and pooled over these 10 imputed datasets. Second, we applied the exclusion criteria (i.e., being nursing home resident, pregnancy, loss to follow-up, and > 30% missing data points on covariates and potential confounders). Third, descriptive statistics were presented. Specifically, amounts and percentages, median and interquartile range, and mean and standard deviation were provided for categorical, non-normally distributed continuous, and normally distributed continuous variables, respectively. Fourth, we used fixed effects models to examine the associations between changes in fast-food outlet exposure and BMI change. Here, BMI change is based on the BMI data at baseline and follow-up. By only considering within-person and not between-person variation, fixed effects models automatically control for all observed (e.g., sex) and unobserved covariates and potential confounders that do *not* change over time. We then used an unadjusted model and a model adjusted for *changes* in the aforementioned covariates and potential confounders between baseline and follow-up. In the fixed effects models, we added cluster-robust standard errors to take into account clustered data within individuals. Namely, two (baseline and follow-up) observations per participant were analysed and these repeated measurements within the same individual tend to cluster. As only *within*-person variation is considered in fixed effects analyses, further addressing clustered data *between* individuals, such as between individuals from different neighbourhoods, is not required. We presented unstandardised effect sizes with 95% confidence intervals. The threshold for statistical significance was set at *p* = 0.05 (for two-sided testing) while we also reported p for trend (*p* < 0.10). All analyses were performed in Rstudio v3.5.2.

To investigate moderation by age and by baseline level of fast-food outlet exposure, we added two-way interaction terms between changes in fast-food outlet exposure and age and two-way interaction terms between changes in fast-food outlet exposure and baseline level of fast-food outlet exposure on BMI change. If at least one these interactions terms had a p-value < 0.10 [42]), we stratified our analyses for participants aged 18–29, 30–39, 40–49, 50–59, and 60 + years, or participants with null, one, or at least two fast-food outlets within 1 km at baseline.

To evaluate robustness of results, we repeated the analyses with waist-to-height ratio as the outcome. Body Mass Index is a common weight status measure because of its quick and inexpensive assessment, yet waist-to-height ratio more adequately reflects fat mass [43] and regional fat distribution [35] and hence is less susceptible to misclassification [44]. Furthermore, we repeated the analyses on a subgroup of participants that did not move houses between the baseline and follow-up assessment (*N* = 80,369). In this subgroup, we expect fewer changes in covariates and potential confounders to occur, as moving houses is also associated with changes in socio-demographic characteristics (e.g., living situation) and environment (e.g. address density).

## Results

### Study population

**Fig. 1:**
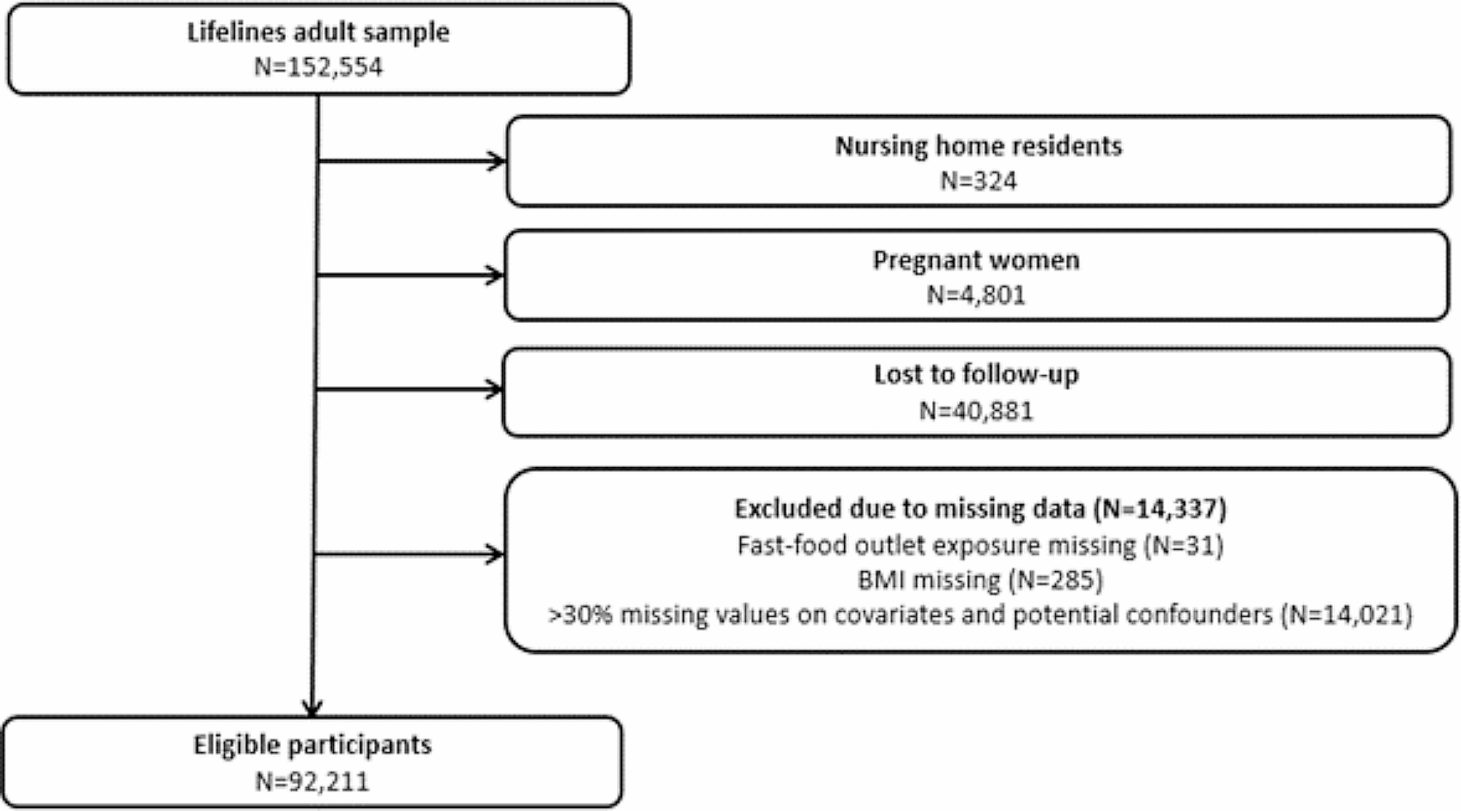
Flowchart of the selection of participants for the current study

The final study population (Fig. 1) consisted of 92,211 eligible participants from 2,894 neighbourhoods. These participants had a mean age of 46.2 (SD: 12.3) years and 57.4% were female (Table 1). The mean BMI was 26.0 (SD: 4.2) kg/m^2^ and 6.2% of participants’ BMI was explained by the neighbourhood they lived in. The median number of fast-food outlets within 1 km of the residential address was 3 (IQR: 1–7). The overall percentage missing data on covariates and potential confounders was 6.3% (percentage and amount of missing data per variable is presented in Table S6). Compared to eligible participants, participants who were lost to follow-up or excluded due to missing data were younger, had a higher BMI at baseline and a lower income, and lived in neighbourhoods of lower socio-economic status (Table S2).

**Table 1:** Baseline characteristics of study population (*N* = 92,211)

Variable	
Age, mean (SD)	46.2 (12.3)
Sex	
Female, N (%)	52,941 (57.4)
Weekly working hours, median (IQR)	24 (0–36)
Years of education received, median (IQR)	12 [10–15]
Household situation	
Living alone, N (%)	9,660 (10.5)
Living together with one household member, N (%)	32,281 (35.1)
Living together with two household members, N (%)	14,081 (15.3)
Living together with three household members, N (%)	24,964 (27.1)
Living together with four household members, N (%)	8,924 (9.7)
Living together with five or more household members, N (%)	2,161 (2.3)
Income, mean (SD)	1,558 (573)
Number of days of at least 30 min of physical activity per week, median (IQR)	5 [3–6]
Pregnancy between baseline and a year before the follow-up assessment, N (%)	4,355 (4.7)
Body Mass Index, mean (SD)	26.0 (4.2)
Change in Body Mass Index, mean (SD)	0.06 (1.73)
Waist-to-height ratio, mean (SD)	0.52 (0.07)
Number of fast-food outlets within 1 km, median (IQR)	3 [1–7]
Change in fast-food outlets within 1 km, median (IQR)	0 (0–1)
Number of physical activity facilities within 1 km, median (IQR)	1 (0–3)
Number of healthy food outlets within 1 km, median (IQR)	2 [1–4]
Neighbourhood address density, in number of addresses per km^2^, median (IQR)	594 (198–1,103)
Neighbourhood socio-economic status, standardised score, mean (SD)	0.03 (0.99)

Note: Characteristics are based on non-imputed data. Percentage represent valid percentages. Note: SD = standard deviation; IQR = interquartile range

### Associations between changes in fast-food outlet exposure and changes in Body Mass Index

Over a mean follow-up period of 3.9 (SD: 1.1) years, participants had a mean BMI change of 0.06 (SD: 1.73) kg/m^2^. Also, 21,322 (23.1%) and 18,216 (18.2%) participants had a BMI *gain* and BMI *loss* of at least 1.0 kg/m^2^, respectively. The median (IQR) change in number of fast-food outlets within 1 km was 0 (0–1). In total, 28,098 (30.5%) and 20,246 (22.0%) participants had an increase or decrease in number of fast-food outlets within 1 km, respectively.

In the adjusted model, *increases* in the number of fast-food outlets within 1 km were associated with BMI increases (Table 2; see Table S3 for effect sizes for fast-food outlet exposure and all covariates and potential confounders on BMI change). For every extra fast-food outlet that emerged between baseline and follow-up, the BMI of participants increased with 0.003 (95% CI: 0.001, 0.006) kg/m^2^. *Decreases* in number of fast-food outlets within 1 km were not associated with BMI change (B (95% CI): 0.001 (-0.001, 0.004)).

**Table 2:** Associations between changes in fast-food outlet exposure and changes in body mass index

Variable	Univariable model Changes in Body Mass Index, B (95% CI)	Multivariable model^1^ Change in Body Mass Index, B (95% CI)
Change in number of fast-food outlets within 1 km		
Increase in number of fast-food outlets within 1 km, per extra fast-food outlet	0.004 (0.002, 0.006)**	0.003 (0.001, 0.006)*
Decrease in number of fast-food outlets within 1 km, per fewer fast-food outlet	0.002 (0.000, 0.004)*	0.001 (-0.001, 0.004)

^1^: Analyses are adjusted for follow-up period, weekly working hours, years of education received, living situation (living alone or together), income, neighbourhood socio-economic status, address density, number of healthy food outlets within 1km, number of physical activity facilities within 1km, pregnancy, and physical activity. *: p-value < 0.05; **: p-value < 0.001

### Moderation analyses

**Fig. 2:**
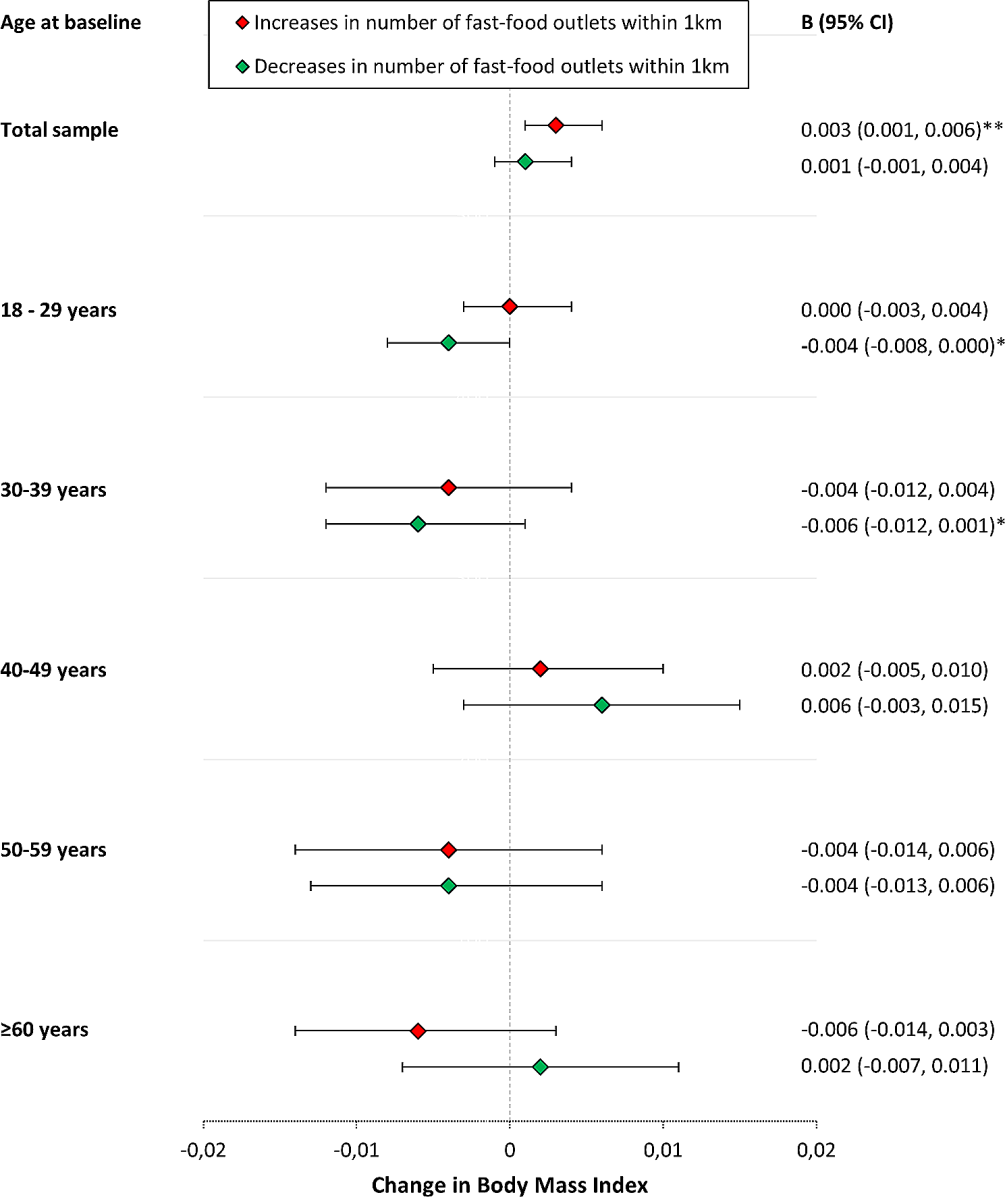
The associations between changes in fast-food outlet exposure and changes in BMI, stratified by age groups. Analyses were adjusted for follow-up period, changes in weekly working hours, years of education received, living situation (living alone or together), income, neighbourhood socio-economic status, address density, number of healthy food outlets within 1 km, number of physical activity facilities within 1 km, pregnancy, and physical activity. *: P-value < 0.10. **: P-value < 0.05

Although the interaction terms between both increases and decreases in fast-food outlet exposure and age on BMI change were significant (*p* < 0.001), a clear moderation pattern was lacking in age-stratified analyses (Fig. 2). Also the associations between increases and decreases in fast-food outlet exposure and BMI change were not present in any of the age subgroups, although a p for trend was observed in adults 18–29 years in the association between decreases in fast-food outlet exposure and BMI changes and in adults 30–39 years in the association between increases in fast-food outlet exposure and BMI changes (Fig. 2). The associations between changes in fast-food outlet exposure and BMI change were not moderated by fast-food outlet exposure at baseline (*p* = 0.52).

### Sensitivity analyses

In sensitivity analyses, changes in fast-food outlet exposure were neither associated with changes in waist-to-height ratio (Table S4) nor with BMI change among participants who did not move houses (*N* = 80,369; Table S4).

## Discussion

We found that increases in the number of fast-food outlets within 1 km of the residential address were associated with BMI increases over approximately four years. Decreases in fast-food outlet exposure within 1 km were not associated with changes in BMI. No clear moderation pattern by age or fast-food outlet exposure at baseline was found in the association between changes in fast-food outlet exposure and BMI changes.

The effect size for increases in number of fast-food outlets within 1 km on BMI increases was 0.003 kg/m^2^ for every extra fast-food outlet that emerged. For a Dutch adult with average height (i.e., 1.75 m [45]), an increase of five fast-food outlets within 1 km would translate into a 46 grams higher weight gain over approximately four years. This effect size could be considered small and not clinically relevant on an individual level. However, at population level, the role of fast-food outlets may be greater: a large proportion of the population may experience an increase in residential fast-food outlet exposure. This may result in a small increase in population mean BMI. A small increase in population mean BMI is important as it may result in substantially more individuals with overweight and obesity [46]. For instance, if the population would experience an increase of one fast-food outlet within 1 km every year for 20 years, the mean BMI of the population would increase from 26.0 to 26.3.

Intriguingly, increases in fast-food outlet exposure were associated with BMI increases, but decreases in fast-food outlet exposure were not associated with BMI loss. Perhaps, increases in fast-food outlets around the residential addresses could increase the awareness of unhealthy food in the environment [30] and social norms that are positive towards fast-food consumption [33]. Such greater awareness of fast-food outlets being present and social norms promoting fast-food consumption may result in greater fast-food consumption. Fast-food consumption, in turn, has been consistently linked with BMI gain [12]. The lack of an association between decreases in fast-food outlet exposure and BMI loss may be explained by that the habit of fast-food consumption remains for some period of time after fast-food outlet exposure is reduced [47]. The detected associations between increases in fast-food outlet exposure and BMI change in this study are not in line with results of previous studies that did not consistently demonstrate any associations between changes in fast-food outlets exposure and BMI change [14–21]. Differences between our results and the results of previous studies may be attributed to differences in study population, eating culture, structure of the built environment, and methodological differences (e.g., adjustment for covariates and potential confounders). Also, we treated increases and decreases in fast-food outlet exposure separately, whereas other studies used these together as a single change score.

Contrary to our hypothesis, the associations between changes in fast-food outlet exposure and BMI change were not stronger in young adults or adults with a lower fast-food outlet exposure at baseline. An explanation for the finding that associations were not stronger in young adults could be that young adults eat their fast-food in other places than the residential environment [48]. Furthermore, metabolism of young adults is faster than metabolism of older people [49]. A potential explanation for the absence of moderation effects of the number of fast-food outlets around the residential address at baseline is that, despite statistical adjustment for neighbourhood socio-economic status, areas with a low baseline level of fast-food outlet exposure are often high neighbourhood socio-economic status areas where inhabitants in general have a healthier lifestyle [50]. Indeed, participants from the top half of neighbourhood socio-economic status areas had a lower number of fast-food outlets at baseline (median (IQR) 1 (0–4)) than participants from the bottom half of neighbourhood socio-economic status areas (median (IQR) 5 [2–12]). Another potential explanation might be that relatively few fast-food outlets opened in areas with a low baseline level of fast-food outlet exposure. Indeed, only 15.0% of participants with null fast-food outlets at baseline had an increase in fast-food outlet exposure, as opposed to 30.5% in the whole sample. The absence of moderation effects by the baseline fast-food environment contrasts with a previous study on Dutch children that found that changes in fast-food outlet exposure were only associated with BMI change in those children who had no fast-food outlet within 400 m at baseline [25]. Perhaps, the difference is due to the fact that the study among children was conducted in an urban setting and different densities were used to measure fast food outlet exposure (400 m versus 1 km).

The sensitivity analyses showed that changes in fast-food outlet exposure were not associated with changes in waist-to-height ratio. This finding may be explained by a lower responsiveness of waist-to-height ratio: Possibly, greater effects of changing fast-food environments would have been needed to detect changes in waist-to-height ratio. The relatively small individual-level effect sizes of changes in fast-food environments in this study would only have led to a change in waist circumference of a few millimetres, which may have been difficult to detect. Also, no associations were found between changes in fast-food outlet exposure and BMI change among participants that did not move houses, perhaps because these participants had less drastic changes in fast-food environment over four years than participants who did move houses.

A strength of this study includes the use of objective BMI measurements, reducing the risk of information bias. Additionally, we used data from a large-scale, representative [27] cohort that covers a large geographical region in the Northern Netherlands, strengthening the generalisability of findings. Further, we assessed the role of *changes* in fast-food outlet exposure in relation to BMI *changes*, whereas previous research mainly relied on cross-sectional and traditional cohort (i.e., the role of single measure fast-food outlet exposure at baseline in relation to BMI changes) designs. Still, this study contains several limitations. Firstly, the follow-up period of approximately four years was relatively short. A longer follow-up period may be needed to capture greater changes in the fast-food environment and to more accurately assess how changes in fast-food outlet exposure results in different dietary habits, and subsequently, BMI change. This could have led to underestimations of effects of changes in fast-food environments on BMI changes in the current study. Secondly, there may be temporal mismatch between measurement of exposure and outcome at baseline (exposure: 2012, BMI: 2006–2013) and follow-up (exposure: 2015, BMI: 2014–2017). Still, most participants had their BMI measured within 1 year from the exposure measurements in 2012 (70.2%) and 2015 (86.3%). Thirdly, we had no data on the actual consumption of fast-food, either by using fast food delivery services and by physically visiting fast-food outelts. Neither do we have data in which circumstances participants eat fast-food. Such data are needed to better understand how changes in fast-food outlet exposure affects visits to fast-food outlets in certain locations and situations, and subsequently BMI change. Additionally, this study was limited to residential fast-food outlet exposure, while fast-food outlet exposure in other places (e.g., the workplace [51]) may also play a role in BMI. Moreover, on average, changes in BMI (mean 0.06 kg/m^2^) and fast-food outlet exposure (median 0) were relatively small. Possibly, observed effects may have been driven by a subset of individuals with greater BMI changes. Finally, even though we could impute missing data points for participants on covariates and potential confounders, the participants that were lost to follow-up or were excluded due to having > 30% missing data points, or missing data on exposure or outcome were younger, had a higher BMI at baseline and a lower income, and lived in neighbourhoods of lower socio-economic status as compared to participants included in the study. This form of attrition bias may have led to underestimated associations.

This study identified increases in fast-food outlet exposure within 1 km of the residential address as a potentially important determinant of BMI increase. This finding should be considered in light of policies targeting the fast-food environment. A recently published study concluded that the Dutch government is not doing enough to improve local food environments, especially targeting fast-food outlets was a particular recommendation [52]. In Northeast England, the policy of discouraging new fast-food outlets to open has been shown to be effective in lowering the proportion and density of fast-food outlets [53]. Other potentially effective policy approaches include restrictions on fast-food marketing [54, 55], and taxations on junk foods [56] and sugar-sweetened beverages [57]. Still, the potential impact of such policy approaches in the Dutch setting should be rigorously evaluated in the future. Also, future studies should investigate the mechanisms and changes in consumer behaviours underlying associations between changes in fast-food outlet exposure and BMI change. Further, the role of fast-food delivery services in BMI change needs to be unravelled. Finally, the role of changes in fast-food outlet exposure in BMI should be unravelled at population level by complex systems approaches. Complex systems approaches can provide insight in the complex interplay between various determinants of BMI, and where in the system interventions are needed to prevent BMI gain [58].

## Conclusions

We identified *increases* in fast-food outlet exposure as a potentially important determinant of BMI increases. Effect sizes were small and not clinically relevant at individual level, but may still be important at population level. Simultaneously, *decreases* in fast-food outlet exposure were not associated with BMI loss.

### Electronic supplementary material

Below is the link to the electronic supplementary material.

Supplementary Material 1

## Data Availability

Data of the Lifelines Cohort Study can be applied for at www.lifelines.nl.

## Abbreviations

BMI: Body Mass Index
IQR: Interquartile range
km: Kilometre
m: Metre
MICEMD: Multiple Imputation by Chained Equations using Multilevel Data
SD: standard deviation
