# Supplementary material for: Effects of changes in residential fast-food outlet exposure on Body Mass Index change: longitudinal evidence from 92,211 Lifelines participants
Source: Int J Behav Nutr Phys Act. 2024 Mar 14;21:31. doi: 10.1186/s12966-024-01577-8 (PMC10941418; doi:10.1186/s12966-024-01577-8)
Supplement: Supplementary file 1 — Supplementary Material 1 [file 12966_2024_1577_MOESM1_ESM.docx]

**Supplementary tables**

**Table S1.** Definitions of fast-food outlets, healthy food outlets, and physical activity facilities.

| **Outlet type** | **Standardised Business Information code** |
| --- | --- |
| **Fast-food outlets** |  |
| Outlets predominantly selling meat products | 47.221 |
| Outlets predominantly selling in bread and banquet | 47.241 |
| Outlets predominantly selling chocolate and confectionary | 47.242 |
| Outlets identified as restaurants or hotel-restaurants containing at least one of the following word elements in their name: ‘pizz’, ‘ijssalon’, ‘snack’, ‘cafetaria’, ‘afhaal’, ‘shoarma’, ‘pannenk’, ‘pannek’, ‘stroop’, ‘wegr’, ‘steak’, ‘rib’, ‘snelbuffet’, ‘venez’, ‘kwalitaria’, ‘alanya’, ‘antalya’, ‘cairo’, ‘shalom’, ‘anatolya’, ‘shaam’, ‘cleopatra’, ‘sahara’, ‘hasret’, ‘ozan’, ‘kebab’, ‘pyramide’, ‘habibi’, ‘cevdet’, ‘ali baba’, ‘babylon’, ‘dönerix’, ‘shaka’, ‘istanbul’, ‘pappa joe’, ‘McDonalds’, ‘Kentucky Fried Chicken’, ‘KFC’, ‘sub’ | 55.101 (hotel-restaurants) and 56.101 (restaurants) |
| Lunch rooms, snack bars, ice cream parlours, and food stalls | 56.102 |
| **Healthy food outlets** |  |
| Supermarkets and similar retail outlets with a general assortment of foods | 47.11 |
| Retail outlets in potatoes, vegetables, and fruit | 47.21 |
| Retail outlets in natural foods and reform articles (e.g., Ekoplaza) | 47.29.2 |
| Marketplace in potatoes, vegetables, and fruit | 47.81.1 |
| Retail outlet selling fish | 47.23 |
| **Physical activity facilities** |  |
| Sport accommodations (e.g., swimming pools) | 93.11 |
| Outdoor sport fields (e.g., athletics court, football court) | 93.12 |
| Gyms | 93.13 |
| Indoor sport facilities (e.g., climbing hall) | 93.14 |
| Water sports (e.g., canoeing facility) | 93.15 |

**Table S2.** Baseline characteristics for eligible participants (N=92,211) and participants excluded due to loss to follow-up or missing data on fast-food outlet exposure, BMI, or >30% of covariates and potential confounders (N=55,218).

| **Variable** | **Eligible participants (N=92,211)** | **Excluded participants (N=55,218)** | **P-value^1^** |
| --- | --- | --- | --- |
| Age, mean (SD) | 46.2 (12.3) | 43.1 (13.9) | <0.001 |
| Sex |  |  |  |
| Female, N (%) | 52,941 (57.4) | 31,302 (56.7) | <0.001 |
| Weekly working hours, median (IQR) | 24 (0 – 36) | 24 (0 – 38) | <0.001 |
| Years of education received, median (IQR) | 12 (10 – 15) | 12 (10 – 15) | <0.001 |
| Household situation |  |  |  |
| Living alone, N (%) | 9,660 (10.5) | 5,629 (11.2) | <0.001 |
| Income, mean (SD) | 1,558 (573) | 1,472 (588) | <0.001 |
| Number of days of at least 30 minutes of physical activity per week, median (IQR) | 5 (3 – 6) | 4 (2 – 6) | <0.001 |
| Pregnancy between baseline and a year before the follow-up assessment, N (%) | 4,355 (4.7) | 1,818 (3.3) | <0.001 |
| Body Mass Index, mean (SD) | 26.0 (4.2) | 26.3 (4.4) | <0.001 |
| Waist-to-height ratio, mean (SD) | 0.52 (0.07) | 0.52 (0.07) | <0.001 |
| Number of fast-food outlets within 1km, median (IQR) | 3 (1 – 7) | 3 (1 – 8) | <0.001 |
| Number of physical activity facilities within 1km, median (IQR) | 1 (0 – 3) | 2 (0 – 3) | <0.001 |
| Number of healthy food outlets within 1km, median (IQR) | 2 (1 – 4) | 2 (1 – 5) | <0.001 |
| Neighbourhood address density, median (IQR) | 594 (198 – 1,103) | 631 (229 – 1,192) | <0.001 |
| Neighbourhood socio-economic status, mean (SD) | 0.03 (0.99) | -0.07 (1.02) | <0.001 |
| Moved houses, N (%) | 14,162 (15.4) | 2,707 (4.9) | <0.001 |

Note: Characteristics are based on non-imputed data. Percentage represent valid percentages. ^1^: P-values are based on two-sample T-tests, Mann-Whitney U tests or Chi-Square tests where relevant.

**Table S3.** Associations between changes in fast-food outlet exposure and changes in Body Mass Index.

| **Variable** | **Univariable model Changes in Body Mass Index, B (95% CI)** | **Multivariable model^1^ Change in Body Mass Index, B (95% CI)** |
| --- | --- | --- |
| **Changes in number of fast-food outlets within 1km** |  |  |
| Increase in number of fast-food outlets within 1km, per extra fast-food outlet | 0.004 (0.002, 0.006)*** | 0.003 (0.001, 0.006)* |
| Decrease in number of fast-food outlets within 1km, per fewer fast-food outlet | 0.002 (0.000, 0.004)* | 0.001 (-0.001, 0.004) |
| **Covariates and potential confounders** |  |  |
| Follow-up period, in months | 0.005 (0.004, 0.006)*** | 0.005 (0.004, 0.005)*** |
| Weekly working hours, per hour | 0.003 (0.002, 0.004)*** | 0.002 (0.001, 0.002)*** |
| Years of education received | 0.048 (0.032, 0.065)*** | 0.040 (0.019, 0.060)*** |
| Living together (reference: living alone) | 0.199 (0.147, 0.250)*** | 0.208 (0.157, 0.260)*** |
| Income, per 100 euros per month | 0.004 (-0.002, 0.010) | 0.003 (-0.002, 0.008) |
| Neighbourhood socio-economic status | -0.032 (-0.057, -0.008)* | -0.034 (-0.061, -0.008)* |
| Address density, per 100 addresses/km^2^ | 0.002 (0.000, 0.005) | 0.000 (-0.004, 0.003) |
| Number of healthy food outlets within 1km | 0.002 (-0.004, 0.008) | -0.004 (-0.013, 0.005) |
| Number of physical activity facilities within 1km | 0.013 (0.005, 0.022)** | 0.010 (0.000, 0.020)* |
| Pregnancy occurred after the first assessment and more than a year before the second assessment (reference: no pregnancy occurred) | 0.098 (0.050, 0.146)*** | 0.068 (0.018, 0.118)** |
| Physical activity, in days per week | -0.023 (-0.028, -0.018)*** | -0.022 (-0.028, -0.016)*** |

*: p < 0.05; **: p < 0.01; ***: p < 0.001; ^a^: p < 0.10

**Table S4.** Sensitivity analyses: repetition of the analyses on (1) changes in waist-to-height ratio instead of changes in BMI as outcome, and (2) a subgroup of participants who did not move houses (N=80,369).

| **Variable** | **Changes in waist-to-height ratio as outcome, B (95% CI)^1^** | **Subgroup analysis on non-movers (B (95% CI)^1^** |
| --- | --- | --- |
| **Exposure** |  |  |
| Increase in number of fast-food outlets within 1km, per extra fast-food outlet | 0.000 (0.000, 0.000) | 0.001 (-0.006, 0.007) |
| Decrease in number of fast-food outlets within 1km, per fewer fast-food outlet | 0.000 (0.000, 0.000) | 0.002 (-0.004, 0.008) |

^1^: Analyses are adjusted for follow-up period, changes in weekly working hours, years of education received, living situation (living alone or together), income, neighbourhood socio-economic status, address density, number of healthy food outlets within 1km, number of physical activity facilities within 1km, pregnancy, and physical activity. *: p-value < 0.05; **: p-value < 0.01; ***: p-value < 0.001; ^a^: p < 0.10.

**Table S5.** STROBE Statement—Checklist of items that should be included in reports of ***cohort studies***

|  | | | Item No | Recommendation | Page No |
| --- | --- | --- | --- | --- | --- |
| **Title and abstract** | | | 1 | (*a*) Indicate the study’s design with a commonly used term in the title or the abstract | 1, 2 |
|  |  |  |  | (*b*) Provide in the abstract an informative and balanced summary of what was done and what was found | 2,3 |
| Introduction | | | | | |
| Background/rationale | | | 2 | Explain the scientific background and rationale for the investigation being reported | 3-5 |
| Objectives | | | 3 | State specific objectives, including any prespecified hypotheses | 4,5 |
| Methods | | | | | |
| Study design | | | 4 | Present key elements of study design early in the paper | 6,7 |
| Setting | | | 5 | Describe the setting, locations, and relevant dates, including periods of recruitment, exposure, follow-up, and data collection | 6,7 |
| Participants | | | 6 | (*a*) Give the eligibility criteria, and the sources and methods of selection of participants. Describe methods of follow-up | 6,7 |
|  |  |  |  | (*b*) For matched studies, give matching criteria and number of exposed and unexposed |  |
| Variables | | | 7 | Clearly define all outcomes, exposures, predictors, potential confounders, and effect modifiers. Give diagnostic criteria, if applicable | 7-9 |
| Data sources/ measurement | | | 8* | For each variable of interest, give sources of data and details of methods of assessment (measurement). Describe comparability of assessment methods if there is more than one group | 7-9 |
| Bias | | | 9 | Describe any efforts to address potential sources of bias | 9, 10 |
| Study size | | | 10 | Explain how the study size was arrived at | 6, 11 |
| Quantitative variables | | | 11 | Explain how quantitative variables were handled in the analyses. If applicable, describe which groupings were chosen and why | 9,10 |
| Statistical methods | | | 12 | (*a*) Describe all statistical methods, including those used to control for confounding | 9,10 |
|  |  |  |  | (*b*) Describe any methods used to examine subgroups and interactions | 9,10 |
|  |  |  |  | (*c*) Explain how missing data were addressed | 9 |
|  |  |  |  | (*d*) If applicable, explain how loss to follow-up was addressed | NA |
|  |  |  |  | (*e*) Describe any sensitivity analyses | 10 |
| Results | | | | |  |
| Participants | | | 13* | (a) Report numbers of individuals at each stage of study—eg numbers potentially eligible, examined for eligibility, confirmed eligible, included in the study, completing follow-up, and analysed | 10,11 |
|  |  |  |  | (b) Give reasons for non-participation at each stage | 10,11 |
|  |  |  |  | (c) Consider use of a flow diagram | 10 |
| Descriptive data | | | 14* | (a) Give characteristics of study participants (eg demographic, clinical, social) and information on exposures and potential confounders | 10,11 |
|  |  |  |  | (b) Indicate number of participants with missing data for each variable of interest | 10 |
|  |  |  |  | (c) Summarise follow-up time (eg, average and total amount) | 12 |
| Outcome data | | | 15* | Report numbers of outcome events or summary measures over time | 12 |
| Main results | 16 | (*a*) Give unadjusted estimates and, if applicable, confounder-adjusted estimates and their precision (eg, 95% confidence interval). Make clear which confounders were adjusted for and why they were included | | | 12 |
|  |  | (*b*) Report category boundaries when continuous variables were categorized | | | 12 |
|  |  | (*c*) If relevant, consider translating estimates of relative risk into absolute risk for a meaningful time period | | | NA |
| Other analyses | 17 | Report other analyses done—eg analyses of subgroups and interactions, and sensitivity analyses | | | 13 |
| Discussion | | | | | |
| Key results | 18 | Summarise key results with reference to study objectives | | | 14 |
| Limitations | 19 | Discuss limitations of the study, taking into account sources of potential bias or imprecision. Discuss both direction and magnitude of any potential bias | | | 16 |
| Interpretation | 20 | Give a cautious overall interpretation of results considering objectives, limitations, multiplicity of analyses, results from similar studies, and other relevant evidence | | | 14, 17 |
| Generalisability | 21 | Discuss the generalisability (external validity) of the study results | | | 14 |
| Other information | | | | | |
| Funding | 22 | Give the source of funding and the role of the funders for the present study and, if applicable, for the original study on which the present article is based | | |  |

*Give information separately for exposed and unexposed groups.

**Note:** An Explanation and Elaboration article discusses each checklist item and gives methodological background and published examples of transparent reporting. The STROBE checklist is best used in conjunction with this article (freely available on the Web sites of PLoS Medicine at http://www.plosmedicine.org/, Annals of Internal Medicine at http://www.annals.org/, and Epidemiology at http://www.epidem.com/). Information on the STROBE Initiative is available at http://www.strobe-statement.org.

**Table S6.** Amount and percentage of missing data points, per variable in the analytical sample. The total percentage of missing data points on covariates and potential confounders in the study was 6.3%.

| **Variable** | **N (%) missing data points** |
| --- | --- |
| Age | 0 (0.0) |
| Sex | 0 (0.0) |
| Weekly working hours | 781 (0.8) |
| Years of education received | 3,413 (3.7) |
| Household situation | 874 (0.9) |
| Income | 50,288 (54.5) |
| Number of days of at least 30 minutes of physical activity per week | 5,002 (5.4) |
| Pregnancy between baseline and a year before the follow-up assessment | 0 (0.0) |
| Body Mass Index | 0 (0.0) |
| Waist-to-height ratio | 2 (0.0) |
| Number of fast-food outlets within 1km | 0 (0.0) |
| Number of physical activity facilities within 1km | 0 (0.0) |
| Number of healthy food outlets within 1km | 0 (0.0) |
| Neighbourhood address density, in number of addresses per km^2^ | 0 (0.0) |
| Neighbourhood socio-economic status, standardised score | 3,874 (4.2) |

Note: Characteristics are based on non-imputed data. Percentage represent valid percentages. Note: SD = standard deviation; IQR = interquartile range.
